# Host restriction, pathogenesis and chronic carriage of typhoidal *Salmonella*

**DOI:** 10.1093/femsre/fuab014

**Published:** 2021-03-05

**Authors:** Amber J. Barton, Jennifer Hill, Christoph J. Blohmke, Andrew J. Pollard

**Affiliations:** Oxford Vaccine Group, Department of Paediatrics, University of Oxford, Oxford OX3 7LE, UK; National Institute for Health Research (NIHR) Oxford Biomedical Research Centre, Oxford OX4 2PG, UK; Department of Clinical Research, Faculty of Infectious and Tropical Diseases, London School of Hygiene and Tropical Medicine, London WC1E 7HT, UK; Oxford Vaccine Group, Department of Paediatrics, University of Oxford, Oxford OX3 7LE, UK; National Institute for Health Research (NIHR) Oxford Biomedical Research Centre, Oxford OX4 2PG, UK; Oxford Vaccine Group, Department of Paediatrics, University of Oxford, Oxford OX3 7LE, UK; National Institute for Health Research (NIHR) Oxford Biomedical Research Centre, Oxford OX4 2PG, UK; Oxford Vaccine Group, Department of Paediatrics, University of Oxford, Oxford OX3 7LE, UK; National Institute for Health Research (NIHR) Oxford Biomedical Research Centre, Oxford OX4 2PG, UK

**Keywords:** enteric fever, typhoid, *Salmonella* Typhi, *Salmonella* Paratyphi A, bacterial pathogenesis, enteric infection

## Abstract

While conjugate vaccines against typhoid fever have recently been recommended by the World Health Organization for deployment, the lack of a vaccine against paratyphoid, multidrug resistance and chronic carriage all present challenges for the elimination of enteric fever. In the past decade, the development of *in vitro* and human challenge models has resulted in major advances in our understanding of enteric fever pathogenesis. In this review, we summarise these advances, outlining mechanisms of host restriction, intestinal invasion, interactions with innate immunity and chronic carriage, and discuss how this knowledge may progress future vaccines and antimicrobials.

## Teaser

In this review the authors summarise advances in the understanding of enteric fever pathogenesis, outlining mechanisms of host restriction, intestinal invasion, interactions with innate immunity and chronic carriage.

## INTRODUCTION

Typhoid and paratyphoid fever are caused by systemic infection with *Salmonella enterica* serovars Typhi and Paratyphi A, B or C, collectively referred to as enteric fever. Of the 14 million cases of enteric fever per year, 10.9 million are attributed to typhoid, and 3.4 million to paratyphoid (Stanaway *et al*. 2019). Transmitted through faecal contamination of food and water, enteric fever is chiefly endemic in South Asia and Africa. The highest burden is in children under 14 years of age. In 2017, enteric fever was estimated to cause 9.8 million disability-adjusted life years and 136 000 deaths (Stanaway *et al*. 2019).

The species *S. enterica* is classified into over 2000 serovars based on their lipopolysaccharide (LPS) and flagellar antigens (World Health Organization 2007). Many of the virulence factors required for *Salmonella* infection are encoded by clusters of genes known as *Salmonella* pathogenicity islands (SPIs), which vary between serovars (Marcus *et al*. 2000). Enteric fever is most commonly caused by serovars *S*. Typhi and *S*. Paratyphi A. *Salmonella* Paratyphi C can also cause enteric fever, as can strains of *S*. Paratyphi B unable to ferment the organic compound d-tartrate (Pinna, Weill and Peters 2016). Human gastroenteritis and invasive non-typhoidal *Salmonella* disease are most commonly caused by serovars *S*. Typhimurium and *S*. Enteritidis (Gal-Mor, Boyle and Grassl 2014).

**Figure 1.**
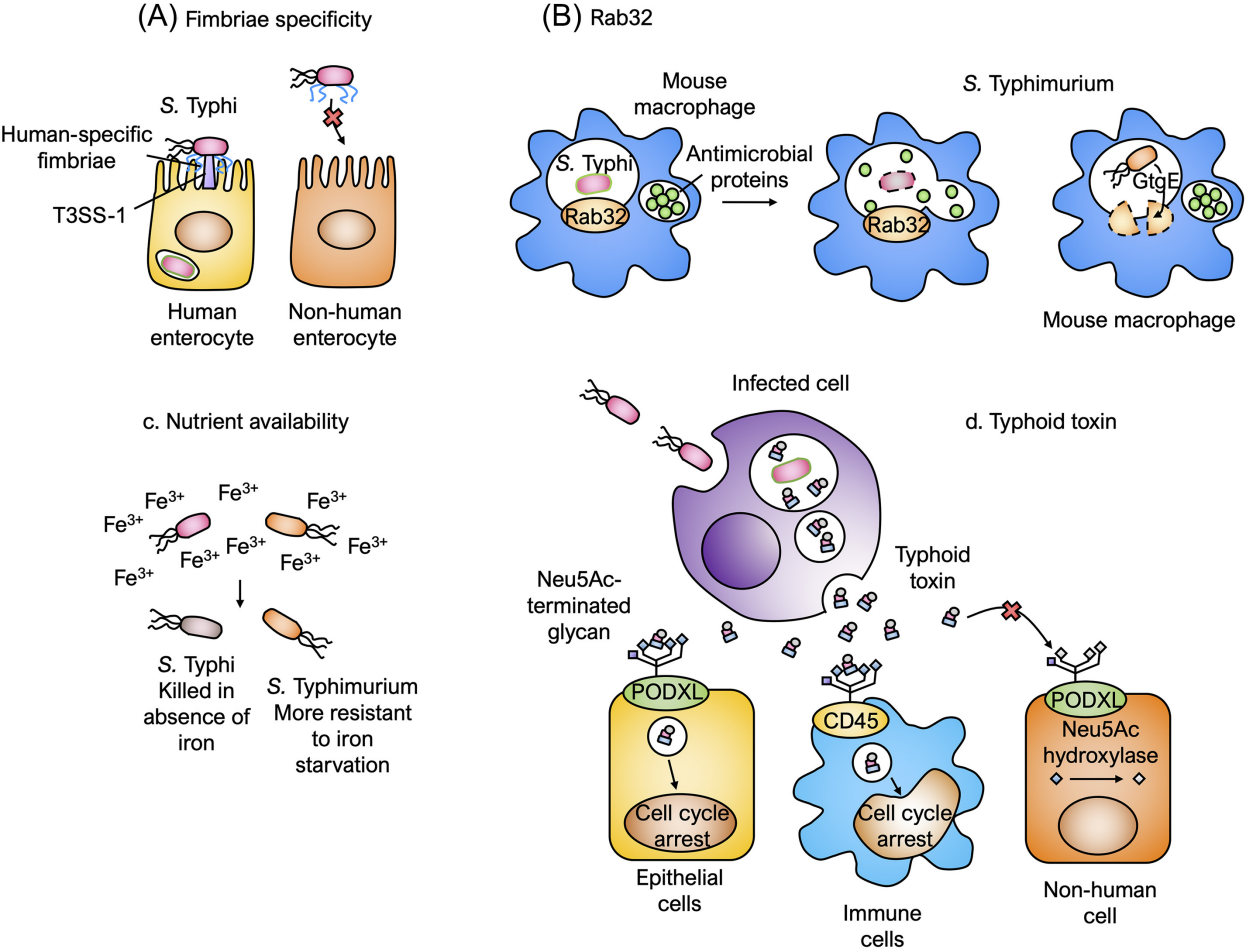
Hypothesised mechanisms of host restriction. **(A)***Salmonella* Typhi fimbriae are specific for adhesion to human epithelial cells, while *S*. Typhimurium fimbriae can adhere to cells from a variety of hosts. **(B)***Salmonella* Typhimurium produces bacterial effector GtgE that cleaves Rab32, preventing killing in mouse macrophages. *Salmonella* Typhi lacks GtgE and is therefore killed in mouse macrophages. **(C)***Salmonella* Typhi is more susceptible to iron starvation than *S*. Typhimurium *in vitro* and in mice, and therefore is only able to infect iron-overloaded mice. **(D)** The typhoid toxin is secreted by intracellular bacteria into the *Salmonella-*containing vacuole and transported to the extracellular space. Here, it binds to target cell receptors (PODXL on epithelial cells, CD45 on macrophages and T and B cells; Galán 2016). The toxin preferentially binds to glycans terminated by Neu5Ac, characteristic of human cells. In the nucleus of target cells, the DNase activity of the toxin causes DNA damage and cell cycle arrest.

Unlike *S*. Typhi, *S*. Typhimurium is not human restricted, and is able to disseminate systemically in orally challenged mice. It has therefore frequently been used as a model of enteric fever (Higginson, Simon and Tennant 2016). In 2010, there were few clues as to why *S*. Typhi is restricted to infecting humans. While a high number of pseudogenes were known to be characteristic of host-restricted *Salmonella* genomes (McClelland *et al*. 2004), and the capsular Vi polysaccharide produced by *S*. Typhi to be immunosuppressive (Wilson *et al*. 2008), mechanisms by which host adaptation modifies host–pathogen interactions have only recently been coming to light. These mechanisms will be discussed in more detail in later sections and are summarised in Fig. 1 and Table 1. Furthermore, as each of the typhoidal serovars underwent genome degradation independently (Didelot *et al*. 2007; Pinna, Weill and Peters 2016; Nair *et al*. 2020), clues as to how *S*. Paratyphi A is able to cause systemic disease are only now emerging (Hiyoshi *et al*. 2018).

**Table 1.** Summary of the evidence as to why *S*. Typhi is invasive in humans but not mice, and why *S*. Typhimurium is invasive in mice but not humans.

	Mice	Humans
*S*. Typhi	Non-invasive	Invasive
	*S*. Typhi lacks gtgE to break down Rab32 in mice	The action of the typhoid toxin is human specific
	*S*. Typhi is susceptible to iron starvation in mice	*S*. Typhi FimH specifically binds human cells
		*S*. Typhi evades innate immune responses at the intestinal epithelium
		*S*. Typhi counters Rab32 in humans using the SPI-1-encoded type III secretion system
*S*. Typhimurium	Invasive	Non-invasive
	*S*. Typhimurium is less susceptible to iron starvation	*S*. Typhimurium FimH only weakly binds human cells
	*S*. Typhimurium produces gtgE to break down Rab32	Induces an innate immune response at the intestinal epithelium and inflammatory diarrhoea

The past decade has seen huge advances in our understanding of the pathogenesis of enteric fever. Particularly important have been development of *in vitro* typhoid models and human experimental infection challenge studies, overcoming the limitation of using the genetically distinct *S*. Typhimurium as a model pathogen. These research efforts have been paralleled by urgent vaccine development to control the disease, culminating in recommendations by the World Health Organization to deploy new typhoid conjugate vaccines in high-burden regions of the world. In this review, we discuss advances in the underpinning biology, including putative mechanisms of host restriction, the means by which typhoidal serovars manipulate innate immunity to disseminate systemically, chronic carriage and the implications for human health.

## GASTROINTESTINAL INVASION

### Anatomical barriers to infection

Upon ingestion of contaminated food or water by a human host, *S*. Typhi faces hostile conditions in the stomach and small intestinal lumen before invading the terminal ileum (Dougan and Baker 2014). The very first barrier to infection is gastric acid. While a high proportion of bacteria are likely killed in the stomach, for those that do survive, the exposure to acid may signal arrival in a new host, acting as a stimulus for *S*. Typhi replication (Ahirwar *et al*. 2014).

The human gut microbiota, consisting of commensal bacteria colonising the gut lumen and mucosa, is thought to play a key role in defence against pathogen invasion. Unlike *S*. Typhimurium, which is able to metabolise butyrate produced by the microbiota and colonise the intestinal lumen, *S*. Typhi and *S*. Paratyphi A lack the required operon (Bronner *et al*. 2018). Despite this, stools rich in transcripts from methane-producing archaea have been associated with a marginally lower risk of developing typhoid after human challenge, suggesting that the microbiome may play a role in enteric fever susceptibility (Zhang *et al*. 2018).

*Salmonella* Typhi must traverse the bactericidal layer of mucus coating the intestinal wall. Mucus predominantly consists of highly glycosylated proteins called mucins (Johansson and Hansson 2016). This layer changes dynamically in response to infection to strengthen the barrier; *S*. Typhi infection induces upregulation of secreted gel-forming mucins *MUC2* and *MUC5B* in an intestinal co-culture model and *ex vivo* intestinal biopsies, respectively (Nickerson *et al*. 2018; Salerno-Goncalves *et al*. 2018). In addition to acting as a barrier, the mucus is enriched with antimicrobial peptides, including α-defensins HD5 and HD6, and human β-defensins 1 and 2. In intestinal biopsies from human volunteers administered *S*. Typhi vaccine strain Ty21a, HD5 messenger RNA (mRNA) was downregulated, potentially representing a means of immune evasion (Simuyandi and Kapulu 2016). However, β-defensins, shown to be bactericidal against *S*. Typhi *in vitro*, and protective in mice infected intraperitoneally with *S*. Typhi (Maiti *et al*. 2014), were unchanged. The bactericidal activity of β-defensins against *S*. Typhi suggests that antimicrobial peptides could have potential as a therapy for antibiotic-resistant enteric fever. In a Phase 3 trial, antimicrobial peptide surotomycin has been demonstrated as non-inferior to vancomycin for *Clostridium difficile* treatment, indicating that such an approach could be effective against gastrointestinal infections (Daley *et al*. 2017). Such a treatment could hypothetically be used as a prophylactic in outbreak settings or reduce transmission through stool shedding.

In addition to antimicrobial peptides, secretory immunoglobulin A (IgA) is thought to protect the intestinal epithelium by blocking bacterial uptake and inhibiting flagellar motility (Betz *et al*. 2018). The role of secretory IgA in protection against enteric fever is not yet well characterised. In human challenge participants, a drop in peripheral B cells and a rise in B cell α4β7 expression are observed during acute disease, suggestive of gut homing (Toapanta *et al*. 2016), while oral vaccination with attenuated *S*. Typhi strain Ty21a significantly increases *S*. Typhi-specific stool IgA (Arya and Agarwal 2014). Serum Vi IgA is associated with protection in Vi-polysaccharide-vaccinated human challenge participants (Dahora *et al*. 2019), but no relationship has been elucidated between gut IgA and protection against enteric fever. Interestingly, mice lacking the polymeric immunoglobulin receptor required for IgA transport to the intestinal lumen are significantly more resistant to *S*. Typhimurium infection (Betz *et al*. 2018), although the potential reasons for this are numerous—possibly due to changes in the microbiome, compensation by serum antibodies or IgG, or reduced M-cell-mediated uptake—and may act in combination.

Underneath the mucus layer, the glycocalyx, composed of glycolipids and glycoproteins extending from the plasma membrane of intestinal epithelial cells, serves as an additional protective barrier. *Salmonella* Typhimurium uses two glycosyl hydrolases, nanH and malS, to degrade the glycocalyx, without which it is cannot efficiently invade (Arabyan *et al*. 2016). While the typhoidal serovars do not possess nanH, malS is present in *S*. Typhi, *S*. Paratyphi A and *S*. Paratyphi B with over 97% sequence identity (UniProt 2019).

### Invasion of the intestinal epithelium

**Figure 2.**
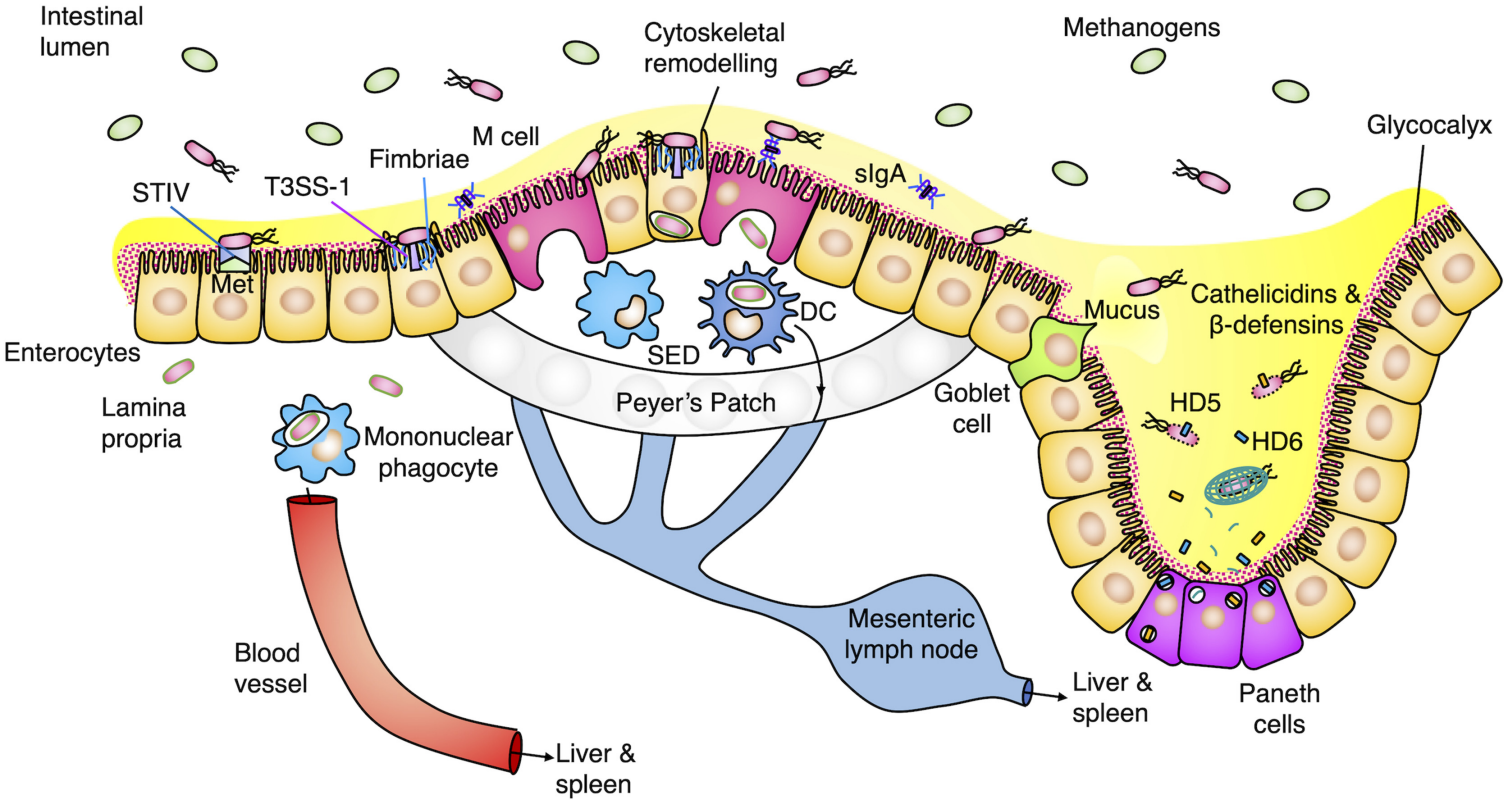
Invasion of the intestinal epithelium by *S*. Typhi. *Salmonella* Typhi invasion is impeded by mucus and the glycocalyx acting as physical barriers, as well as the action of defensins, cathelicidins and IgA in the intestinal mucosa. Those bacteria that successfully adhere to the epithelium using fimbriae cross by T3SS-1- or STIV-mediated invasion of intestinal epithelial cells. Bacteria in the lamina propria and subepithelial dome (SED) are then phagocytosed and systemically disseminated to the liver and spleen via the blood and lymph.

At the surface of the intestinal epithelium, *S*. Typhi attaches to epithelial cells using fimbriae (Berrocal *et al*. 2015) (Fig. 2), extracellular protein structures that mediate bacterial adhesion to epithelial cells. Fimbriae may play a role in host specificity: recombinant *Escherichia coli* expressing *S*. Typhi fimH is only able to strongly bind human epithelial cells, whereas *E. coli* expressing fimH from porcine-, bovine- and avian-adapted *Salmonella* serovars specifically binds cells from their respective hosts (Yue *et al*. 2017). *Escherichia coli* expressing *S*. Typhimurium fimH weakly binds cells from a variety of hosts at equal affinity, possibly reflecting its niche as a non-invasive broad-specificity serovar. While fimH is shared with high homology between *Salmonella* serovars, including *S*. Paratyphi A, B and C, single-nucleotide polymorphisms in this gene are sufficient to change host-specific adhesion (Yue *et al*. 2017; UniProt 2019).

**Figure 3.**
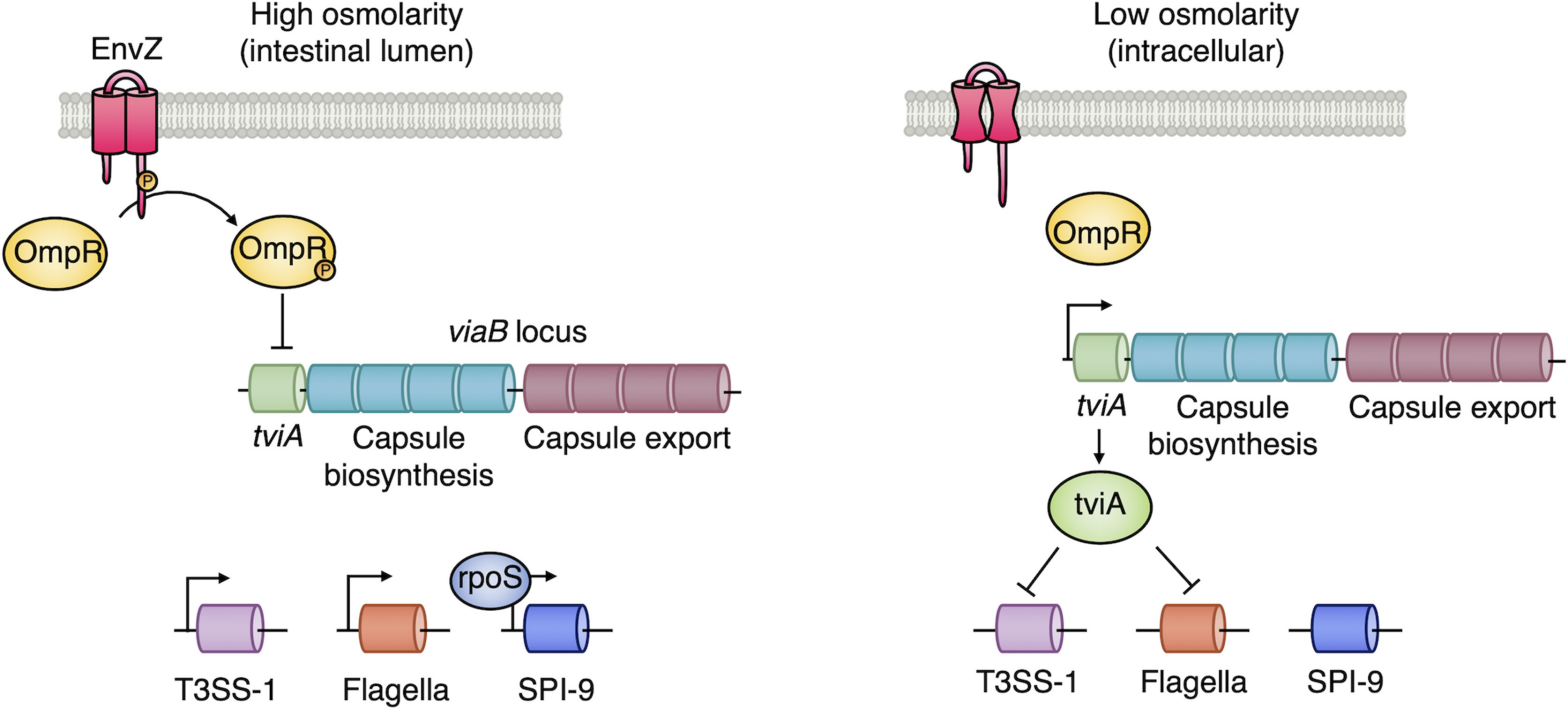
Genetic regulation in response to changes in osmolarity. In the high osmolarity intestinal lumen, EnvZ auto-phosphorylation activity is high, ultimately resulting in the phosphorylation of OmpR and suppression of the *viaB* locus. Thus, Vi capsule biosynthesis is supressed, while T3SS-1 and flagellin expression can take place. In the lower osmolarity environment inside cells, EnvZ undergoes a conformational change that reduces auto-phosphorylation. The *viaB* locus is hence expressed, resulting in synthesis of TviA and the Vi capsule. Regulatory protein TviA then goes on to supress T3SS1 and flagellin expression. Under high osmolarity conditions, SPI-9 is also transcribed by a mechanism dependent on rpoS.

In the high osmolarity environment of the intestinal lumen, the bacterial sensor kinase EnvZ phosphorylates the downstream regulator OmpR (Nuccio, Rüssmann and Bäumler 2010). This in turn supresses *S*. Typhi expression of TviA, a key regulatory protein encoded within the *viaB* operon of the serovar-specific SPI-7 locus (Nuccio, Rüssmann and Bäumler *et al*. 2010) (Fig. 3). Low levels of TviA result in expression of type III secretion system 1 (T3SS1), a needle-like protein complex used to inject *Salmonella* effectors into the host cytosol, and flagellin, thus allowing invasion of the intestinal epithelium (Nuccio, Rüssmann and Bäumler 2010). High osmolarity also induces *S*. Typhi to express SPI-9, enhancing adherence to epithelial cells (Velásquez *et al*. 2016). It has long been thought that *S*. Typhi targets specialised antigen-sampling epithelial cells known as M cells for invasion (Dougan and Baker 2014). However, it lacks the long polar fimbriae used by *S*. Typhimurium to recognise M cells, and in fact appears to preferentially invade enterocytes in a co-culture model and in *ex vivo* biopsies (Gonzales, Wilde and Roland 2017; Nickerson *et al*. 2018). Nonetheless, this question is difficult to resolve fully due to the high numbers of enterocytes in the intestinal epithelium relative to M cells, and the inability to follow gastrointestinal infection *in vivo* in humans.

The chief mechanism of *Salmonella* epithelial invasion is mediated by T3SS-1, a molecular needle encoded by the relatively conserved SPI-1 locus (Sabbagh *et al*. 2010). *Salmonella* Typhimurium injects effector proteins SipA, SipC, SopE and SopE2 into the host cytosol. The effectors activate Rho-family GTPases, small signalling proteins that control cytoskeleton dynamics (Hodge and Ridley 2016). This results in cytoskeletal rearrangement so as to engulf *S*. Typhimurium. Surprisingly, the effector protein SopE2 inhibits epithelial cell invasion (Valenzuela *et al*. 2015). In *S*. Typhimurium the bacterial ubiquitin ligase SopA counteracts the inhibitory effect of SopE2, allowing invasion to take place but also inducing interleukin-8 (IL-8) secretion (Valenzuela *et al*. 2015). In *S*. Typhi both SopA and SopE2 are pseudogenes, allowing it to invade cells while avoiding production of IL-8. *Salmonella* Paratyphi A, however, possesses SopE2 but not SopA (Johnson, Mylona and Frankel 2018). Like *S*. Typhimurium, *S*. Typhi infection induces the enterocyte cytoskeleton to protrude at sites of invasion (Nickerson *et al*. 2018). Reacting to invasion by *S*. Typhi, enterocytes downregulate genes involved in cytoskeleton remodelling (Nickerson *et al*. 2018). This may represent a host response to prevent further infection, as pharmacological inhibition of actin or microtubule assembly almost entirely blocks *S*. Typhi uptake.

To avoid excessive Rho-family GTPase stimulation, which would lead to the activation of NF-κB and inflammatory gene expression (Winter *et al*. 2014), *S*. Typhimurium effector SptP reverses these changes to the cytoskeleton, while the effector AvrA blocks nuclear translocation of NF-κB subunit p65 (Collier-Hyams *et al*. 2002). In *S*. Typhi, however, a different strategy is required, as the gene encoding AvrA is absent and its translated SptP protein is too unstable to be secreted. Rather, sensing the drop in osmolarity as it enters the cell, *S*. Typhi upregulates TviA, resulting in concomitant downregulation of T3SS-1 (Nuccio, Rüssmann and Bäumler 2010). By doing so, excessive Rho-family GTPase stimulation is avoided, preventing the associated induction of NF-κB signalling (Winter *et al*. 2014). It is unclear which of these strategies is more successful: whereas *S*. Typhi induces greater NF-κB responses than *S*. Typhimurium in infected Henle-407 cells, in *ex vivo* infected intestinal biopsies the opposite appears to be true (Hannemann and Galán 2017; Nickerson *et al*. 2018). Unlike *S*. Typhimurium, *S*. Typhi does not induce activation of STAT3, a transcription factor that upregulates IL-10 in response to *S*. Typhimurium infection (Hannemann and Galán 2017; Jaslow *et al*. 2018). This is likely due to the lack of SopE2 (Ruan *et al*. 2017) and SarA (Jaslow *et al*. 2018) in *S*. Typhi. Although T3SS-1-dependent invasion is well characterised, *S*. Typhi is also able to invade epithelial cells via bacterial outer membrane protein STIV, binding the receptor tyrosine kinase Met to induce uptake (Chowdhury *et al*. 2019). Both the typhoidal and non-typhoidal *Salmonella* serovars possess STIV (UniProt 2019).

Having passed through the epithelium, *S*. Typhimurium flagellin activates toll-like receptor (TLR)-5 at the basolateral surface, giving rise to neutrophil recruitment and inflammatory diarrhoea (Keestra-Gounder, Tsolis and Bäumler 2015). In *S*. Typhi, however, the additional role of TviA in mediating downregulation of flagellin limits TLR-5 recognition, as well as activating biosynthesis of the protective Vi polysaccharide capsule. Infection of human colonic explants with wild-type *S*. Typhi elicits lower levels of IL8 expression relative to a Δ*tviA S*. Typhi strain (Raffatellu *et al*. 2005). It may be this upregulation of the Vi capsule as the bacterium crosses the intestinal barrier that renders it immediately susceptible to immunoglobulins induced by Vi-based typhoid vaccines.

Although *S*. Typhi is able to pass through the epithelium without causing inflammatory diarrhoea, there is evidence to suggest a host cytokine response occurs at this point. In a co-culture model of the intestinal epithelium, IL1β, IL17A, TNF-α, IL6, CCL3 and IL8 were released in response to *S*. Typhi infection (Salerno-Goncalves *et al*. 2019). Although secretion of TNF-α, IL6 and CCL3 was reduced in the absence of macrophages, of these three cytokines only TNF-α was directly produced by macrophages in response to stimulation, suggesting that macrophages may modulate cytokine secretion by other cells in the intestinal mucosa. *Salmonella* Paratyphi B induced greater secretion of IL6 and TNF-α than *S*. Paratyphi A or *S*. Typhi, while *S*. Paratyphi A induced greater secretion of CCL3 (Salerno-Goncalves *et al*. 2019). Despite possessing the *viaB* locus, *S*. Typhi still stimulated IL8 secretion from the fibroblasts, endothelial and epithelial cells in the model, as did *S*. Paratyphi A and B (Salerno-Goncalves *et al*. 2018, 2019). This may indicate that IL8 release is dampened down but still significant, or that the Vi capsule was poorly expressed under these culture conditions. Likewise, there was a trend towards apical IL8 secretion by intestinal biopsies in response to *S*. Typhi, as well as significant apical secretion of IL10, IL2 and IL4 (Nickerson *et al*. 2018). However, there was no significant cytokine release at the basolateral side, which could account for the lack of neutrophil infiltration in typhoid fever. Dose-dependent induction of plasma cytokines sCD40L, fractalkine, GROα, IL1RA, EGF and VEGF is observed in typhoid challenge participants within 12 h of challenge, the period in which intestinal invasion is expected to take place; however, the origin of this signal is as yet unconfirmed (Blohmke *et al*. 2016).

This stage of infection likely represents the point at which antibodies induced by mucosal vaccines target invading *S*. Typhi, and there is increasing evidence that parenteral vaccines can also induce protective mucosal responses (Clements and Freytag 2016). Subcutaneous vaccination with STIV has been shown to protect iron-overloaded mice from death following *S*. Typhi and *S*. Paratyphi A challenge, and is therefore an attractive target for a bivalent vaccine. The role of T3SS-1 effector proteins in invasion also renders them potential targets: vaccination with SipD for example, which controls assembly of the T3SS and is shared by *S*. Typhi, *S*. Paratyphi A and *S*. Typhimurium, is capable of protecting orally vaccinated mice against *S*. Typhimurium challenge (Fasciano *et al*. 2019). Likewise, subunit vaccines composed of SipB/SipD or SseB/SseC constructs, effectors shared between serovars, have also proved effective in protecting mice against *S*. Typhimurium challenge (Fasciano *et al*. 2019).

In summary, to invade the intestinal epithelium *S*. Typhi must first survive gastric acid exposure, evade antimicrobial peptides in the mucus and break down the glycocalyx. Upon reaching the epithelium, *S*. Typhi can then use its fimbriae to attach to enterocytes, and induce uptake using T3SS-1 or STIV. To avoid immune activation via Rho-family GTPase stimulation or TLR-5, the TviA locus allows *S*. Typhi to respond to changes in osmolarity by downregulating T3SS-1 and flagellin, and upregulating the immunomodulatory Vi capsule.

## INTERACTION WITH INNATE HOST DEFENCES

### Dissemination via mononuclear phagocytes

Although *S*. Typhi DNA has been detected in the blood of human challenge participants in the first 24 h following challenge (Darton *et al*. 2017), there is currently no direct evidence to show how *S*. Typhi disseminates systemically to the spleen and liver in humans. Mouse models using *S*. Typhimurium suggest dissemination is mediated by migration of infected mononuclear phagocytes from the intestine into the blood or lymph (Vazquez-Torres *et al*. 1999). Pseudogenisation of bacterial effector SseI, which has occurred in both *S*. Typhi and strains of the invasive *S*. Typhimurium sequence type ST313, enhances systemic dissemination of *S*. Typhimurium in orally challenged mice due to increased uptake by CD11b^+^ migratory dendritic cells (Carden *et al*. 2017).

In order to achieve dissemination within mononuclear phagocytes, *S*. Typhi must evade detection and killing by these cells. Although *S*. Typhi can be phagocytosed, uptake of *S*. Typhi and *S*. Paratyphi A is reduced in the absence of functional flagella (Elhadad *et al*. 2015; Schreiber *et al*. 2015) and fimbriae (Berrocal *et al*. 2015), suggesting that phagocytes can also be actively invaded. During entry to these cells the *S*. Typhi Vi capsule has a variety of immunomodulatory effects. Vi masks LPS from recognition by TLR-4 on the cell surface, preventing subsequent release of inflammatory cytokines TNF-α, IL6 or IL8 (Wilson *et al*. 2008), and reduces phagocytosis-mediated by BPI, an antimicrobial protein that binds LPS (Balakrishnan, Schnare and Chakravortty 2016). Despite the absence of Vi, the percentage of IL8^+^ macrophages following stimulation with *S*. Paratyphi A and B is no greater than after stimulation with *S*. Typhi, suggesting that these serovars may possess alternative mechanisms to suppress IL8 secretion (Salerno-Goncalves *et al*. 2019). For example, although *S*. Paratyphi A LPS is exposed and capable of binding TLR-4, it does not activate it, acting as a competitive TLR-4 inhibitor in the presence of *S*. Typhimurium LPS (Chessa *et al*. 2014).

Having formed a vacuole within the cells, in order to survive *S*. Typhi needs to avoid host-derived reactive oxygen and nitrogen species. In the absence of TLR-4-mediated NF-κB activation the transcription of inducible nitric oxide synthase is reduced, limiting synthesis of bactericidal nitric oxide (Wilson *et al*. 2008). The acidic environment of the *Salmonella* containing vacuole induces expression of SPI-2, allowing *S*. Typhimurium to prevent NADPH oxidase assembly on the phagosome membrane and evade killing (Gallois *et al*. 2001; Liew *et al*. 2019). While SPI-2 deletion does reduce the virulence of *S*. Paratyphi A when injected into the peritoneum of mice, ability to colonise the liver and spleen is retained (Yin *et al*. 2020). In *S*. Typhi, knock out of SPI-2 has no effect on survival within human macrophages (Forest *et al*. 2010). The redundancy could in part be due to deterioration of epithelial invasion regulator *marT* into a pseudogene in *S*. Typhi, creating a new open reading frame that appears to encode a novel H_2_O_2_-protective protein (Ortega *et al*. 2016). Furthermore, a eukaryote-like serine/threonine kinase unique to *S*. Typhi is induced in response to H_2_O_2_, promoting survival within macrophages and contributing to virulence in infected mice (Theeya *et al*. 2015). As inhibitors of eukaryotic serine/threonine kinases are already approved as cancer treatments, the eukaryote-like serine/threonine kinase unique to *S*. Typhi may be an attractive therapeutic target, rendering the bacteria more susceptible to killing by H_2_O_2_ (Kannaiyan and Mahadeva 2017). The *suf* operon, involved in producing Iron-Sulphur clusters under oxidative stress, also appears to contribute to *S*. Typhi survival within macrophages (Wang *et al*. 2015). Within monocyte-derived dendritic cells, *S*. Typhi switches from carbohydrate to lipid consumption (Xu *et al*. 2019). The significance of this is not yet understood, although for *S*. Typhimurium lipid metabolism is necessary for replication within pro-inflammatory macrophages (Reens, Nagy and Detweiler 2020).

Within macrophages, *S*. Typhi must also avoid killing mediated by host protein Rab32 (Spano and Galán 2012). Although the mechanism of killing has not yet been elucidated, Rab32 is involved in the delivery of cargo to lysosome-related organelles, and therefore may allow delivery of antimicrobial proteins to the *Salmonella* containing vacuole (Spano and Galán 2012). While *S*. Typhimurium produces the protease gtgE, allowing it to break down Rab32 and therefore survive in mouse macrophages, *S*. Typhi does not, resulting in rapid killing. In human macrophages, however, *S*. Typhi is instead thought to counter this pathway though a mechanism dependent on its SPI-1-encoded T3SS1 (Baldassarre *et al*. 2019).

Rather than being killed, internalised *Salmonella* can induce macrophage death; however, it is as yet unknown whether this acts to benefit the bacteria or the host. For *S*. Typhimurium, bacterial effector protein SipB activates caspase-1, resulting in a rapid and inflammatory cell death known as pyroptosis (Chen *et al*. 2015). SipB knockout reduces the virulence of *S*. Typhimurium in mice, and is also present in the typhoidal *Salmonella serovars* (Chen *et al*. 2015; UniProt 2019). In addition to direct activation by bacterial effectors, recognition of *S*. Typhimurium flagellin by sensor protein NAIP activates the NLRC4 inflammasome, a multiprotein complex that induces caspase-1 activation (Kortmann, Brubaker and Monack 2015; Winter *et al*. 2015; Brewer, Brubaker and Monack 2019). This suggests that *S*. Typhimurium*-*induced cell death could in fact represent a protective host response. The temporal association between pyroptosis and *S*. Typhimurium clearance has led to the suggestion that bacterial release from dying macrophages leaves *S*. Typhimurium vulnerable to uptake and killing by neutrophils (Miao *et al*. 2010). This has been confirmed by mouse experiments finding that caspase-1 knockout reduces bacterial clearance by neutrophils and increases susceptibility to *S*. Typhimurium (Broz *et al*. 2012). Although *S*. Typhi flagellin is a particularly potent NLRC4 activator (Yang *et al*. 2014), the TviA-dependent downregulation of flagellin appears to reduce NLRC4 activation and pyroptosis, potentially acting as a means of immune evasion (Winter *et al*. 2015). In contrast, typhoidal serovar *S*. Paratyphi A induces a high level of macrophage killing (Salerno-Goncalves *et al*. 2019).

During acute disease the level of iron-regulating hormone hepcidin is significantly raised in the serum, resulting in the sequestration of iron within macrophages (Darton *et al*. 2015). Iron starvation is an important mechanism of host defence, as illustrated by the susceptibility of iron-overloaded mice to *S*. Typhi infection (Das *et al*. 2019). This raises the possibility of sequestering iron from *S*. Typhi as a feasible strategy for treatment. While small molecule iron chelators such as desferrioxamine can be utilised by *S*. Typhi and therefore enhance growth, iron chelating polymers too large to be accessible to bacteria are capable of suppressing *Staphylococcus aureus* wound infections in mice (Parquet *et al*. 2018). While diversion of iron to macrophages in acute disease might restrict the growth of free bacteria, it could be advantageous to bacteria residing intracellularly. As well as being required for bacterial growth, iron activates *S*. Typhi ferric uptake regulator (Fur), repressing sRNAs RfrA and RfrB, and enhancing H_2_O_2_ resistance and intracellular survival through an unknown mechanism (Leclerc, Dozois and Daigle 2013). It has more recently been found that in the case of *S*. Typhimurium infection in Raw264.7 cells, hepcidin increases iron in the cytosol but decreases it in the *Salmonella*-containing vacuole (Lim, Soo Kim and Jeong 2018). Rather than starving *S*. Typhimurium, the lack of iron impairs production of bactericidal reactive oxygen species and results in a higher bacterial load in mice.

To summarise, following invasion *S*. Typhi is thought to be taken up by mononuclear phagocytes, through which it disseminates systemically in a primary bacteraemia. Evasion of killing by upregulating H_2_O_2_-protective proteins and the Vi capsule, and shielding LPS from TLR-4 to prevent iNOS transcription are important to allow *S*. Typhi to survive host defences. By downregulating flagellin expression, *S*. Typhi is able to prevent its host cell undergoing pyroptosis, averting the resultant inflammation and uptake by neutrophils.

### Evading complement-dependent neutrophil activation

**Figure 4.**
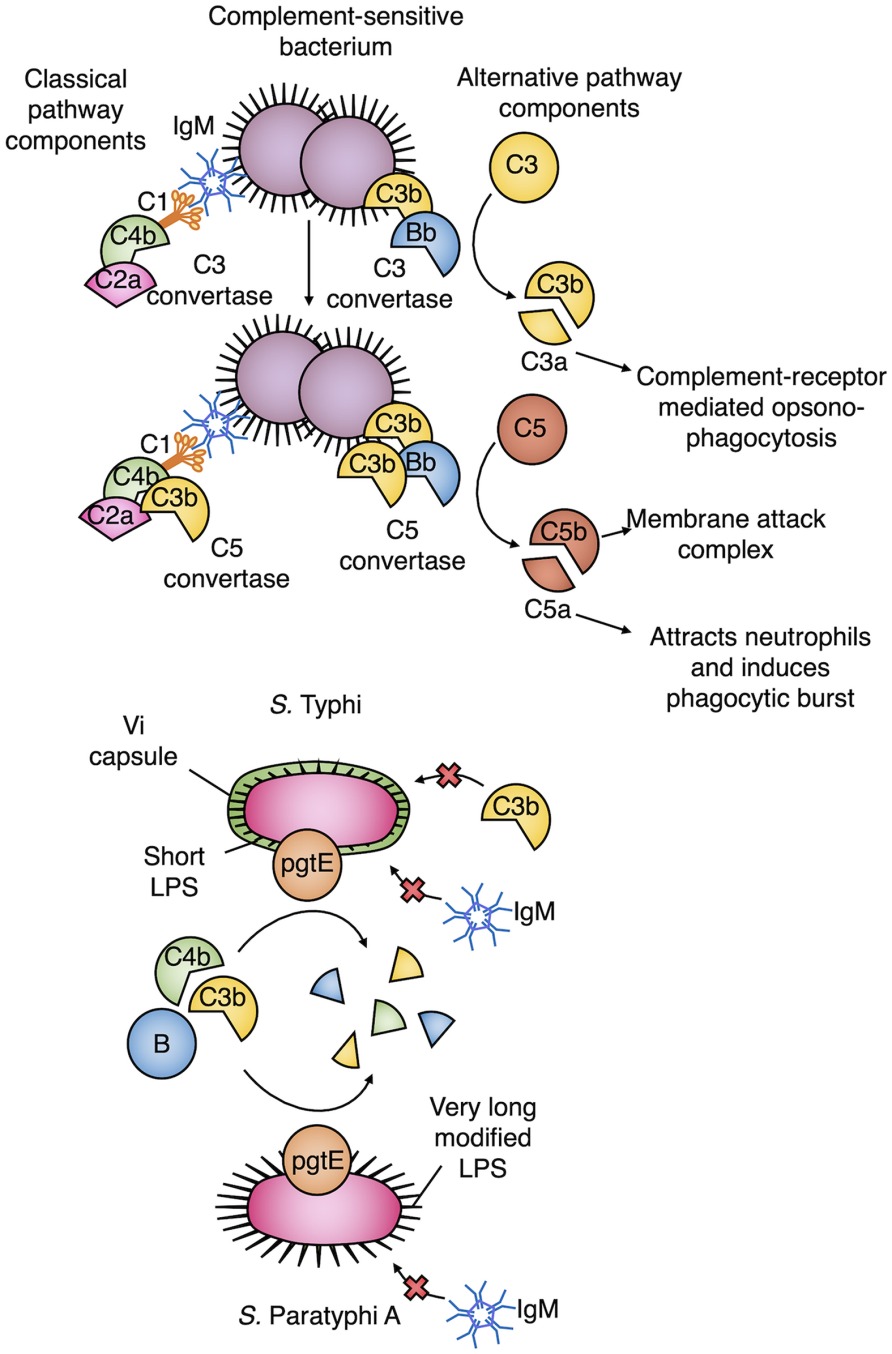
Complement evasion by *S*. Typhi and *S*. Paratyphi A. The alternative and classical complement pathways culminate in the formation of C3 and C5 convertases, resulting in the attraction of neutrophils by C5a and opsonisation by C3b. In *S*. Typhi expression of the Vi capsule and absence of very long O-antigen chains prevents C3b and IgM deposition, while in *S*. Paratyphi A the production of very long modified O-antigens prevents IgM binding shorter O-antigen chains on its surface. Furthermore, the surface protease PgtE cleaves C3b, C4b and B.

The considerable efficacy of the T cell-independent Vi polysaccharide vaccine (Milligan *et al*. 2018) and the association between baseline LPS antibody and resistance to *S*. Paratyphi A in human challenge (Dobinson *et al*. 2017) suggests that at some stages during the course of infection, these typhoidal *Salmonella* bacteria must be extracellular, and therefore vulnerable to opsonisation. However, *S*. Typhi appears to have evolved several mechanisms to evade complement-dependent opsonisation and killing (Fig. 4): shielding by the Vi capsule, LPS modification and breakdown of complement components by PgtE. The lack of free hydroxyl groups in the Vi capsule prevents C3b from binding the bacterial surface (Wilson *et al*. 2011). In the absence of Vi-specific antibodies, the Vi capsule also reduces IgG, C3 and membrane attack complex binding (Hart *et al*. 2016). This protection is enhanced by a nonsense mutation in the *fepE* gene, preventing synthesis of very long O-antigen chains that would expose hydroxyl groups to C3b at the capsule surface (Crawford *et al*. 2013). In addition,*S*. Typhi shares an operon with *S*. Typhimurium that glucosylates the O-antigen on LPS to reduce C3 binding (Riva, Korhonen and Meri 2015). Conserved surface protease PgtE is present in both typhoidal and non-typhoidal serovars, and cleaves C3b, C4b and B (Kintz *et al*. 2017; UniProt 2019). Therapeutically, it is possible that *Salmonella* could be rendered more susceptible to complement by inhibition of the complement-cleaving protease PgtE. *In silico* docking of FDA-approved protease inhibitors suggested that the antiretroviral drug indinavir bound PgtE with the highest affinity (Samykannu *et al*. 2018). However, this finding has not yet been validated experimentally. While *S*. Paratyphi A lacks the Vi capsule and produces functional *fepE*, the O-antigen of *S*. Paratyphi A differs due to the pseudogenisation of *rfbE*, giving it a branching paratose residue that prevents IgM-mediated activation of the classical complement pathway (Hiyoshi *et al*. 2018).

Despite correlating with disease attenuation, serum bactericidal antibody has not been found to associate with protection in the human challenge model to date (Juel *et al*. 2018). This raises the possibility that the direct role of complement in *S*. Typhi lysis is secondary to its role in attracting and activating phagocytes. Inhibition of complement deposition by factors such as the Vi capsule reduces generation of chemoattractant C5a, impairing neutrophil recruitment (Wangdi *et al*. 2014). However, in the presence of vaccination-induced antibodies against the Vi capsule, neutrophil phagocytosis of Vi-coated beads is increased, particularly in participants who remained healthy following subsequent *S*. Typhi challenge (Celina *et al*. 2021). Therefore, vaccination may act to overcome this method of evasion. Although peripheral blood neutrophil counts drop in acute disease, the peripheral blood transcriptome is dominated by clusters associated with neutrophils, suggesting a significant involvement in the immune/inflammatory response (Waddington *et al*. 2014; Blohmke *et al*. 2016). Calprotectin, a chelating protein complex that constitutes 40% of neutrophil cytosol, is raised in both the plasma and faeces of patients with typhoid fever, and is able to inhibit the growth of *S*. Typhi *in vitro* (De Jong *et al*. 2015). As in macrophages, neutrophil phagocytosis of *S*. Typhi or *S*. Paratyphi A fails to stimulate a bactericidal oxidative burst by NADPH oxidase (Hiyoshi *et al*. 2018), although it is not currently clear whether this is sufficient to suppress killing by neutrophils.

Overall, it appears that *S*. Typhi and *S*. Paratyphi A have undergone convergent evolution in order to evade complement binding, preventing chemo-attraction of neutrophils and therefore oxidative killing. The analogous role of *S*. Paratyphi LPS to the Vi capsule in immune evasion supports the pursuit of this antigen as an *S*. Paratyphi A vaccine target. While *S*. Paratyphi C also produces the protective Vi capsule, the mechanism by which typhoidal strains of *S*. Paratyphi B evade complement remains elusive.

### Natural killer cell stimulation

While the role of natural killer (NK) cells is well established in viral and cancer immunity, a contribution to bacterial immunity has come to light more recently. In mice infected with *S*. Typhimurium, IL18-mediated recruitment of NK cells to the gut did not affect bacterial load, but did increase intestinal inflammation (Müller *et al*. 2016). Although gastrointestinal inflammation is not a hallmark of human enteric fever as it is in mice, the percentage of NK cells producing granzyme A does rise (De Jong *et al*. 2017). Furthermore, in participants receiving attenuated oral vaccine *S*. Typhi strain Ty21a, gene sets relating to NK cells were enriched in the whole-blood transcriptome (Blohmke *et al*. 2017). *In vitro* stimulation of NK cells with fixed *S*. Typhi enhanced expression of activation marker CD69 as well as their killing ability, while stimulation with attenuated vaccine strains Ty21a and M01ZH09 increased the proportion of CD107a and interferon-γ-positive NK cells (Puente *et al*. 2000; Blohmke *et al*. 2017). However, it is yet to be determined whether NK cells play a role in immunity to enteric fever *in vivo*.

## THE CARRIER STATE

**Figure 5.**
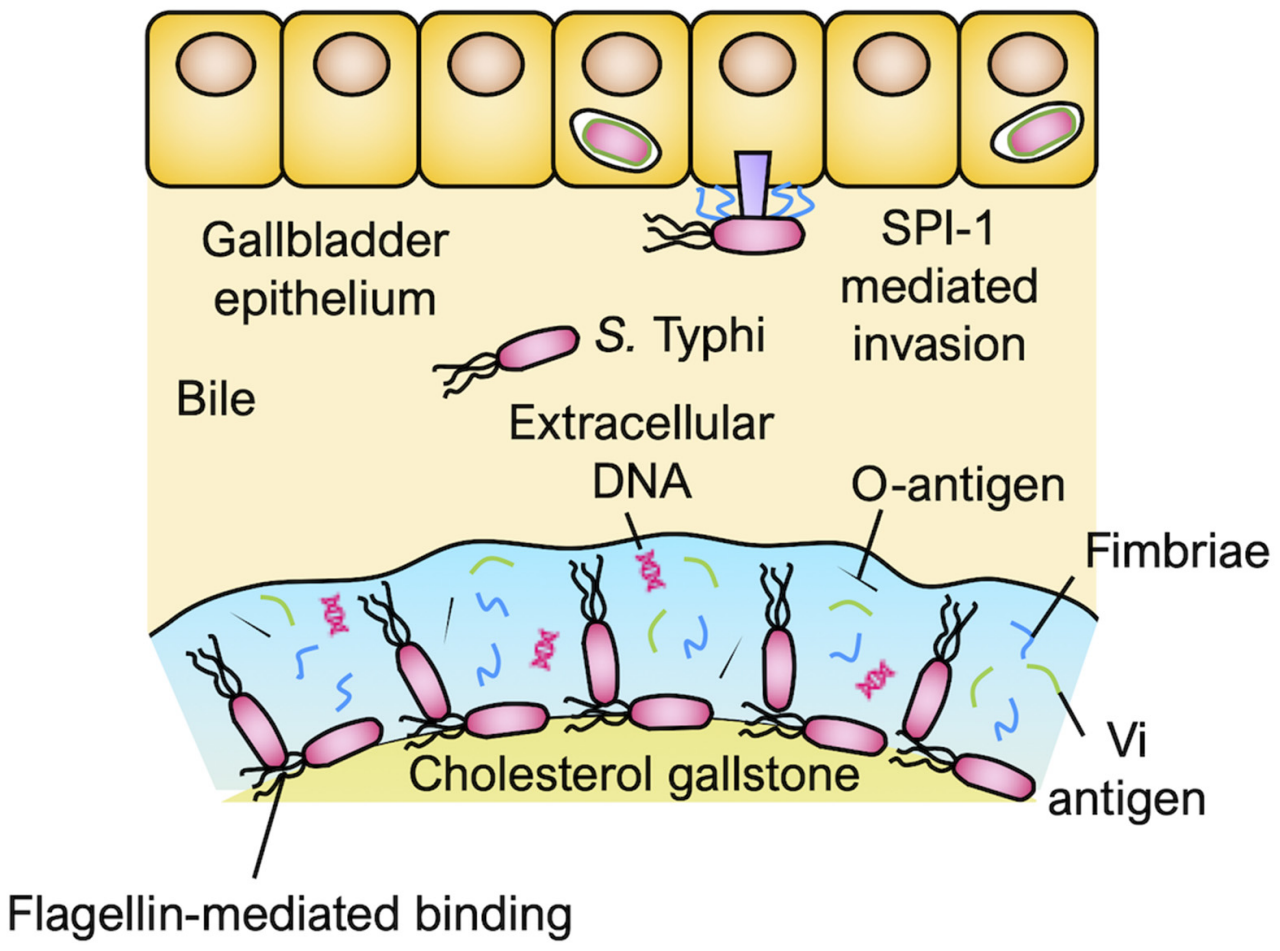
Chronic carriage of *S*. Typhi in the gallbladder. Bile induces *S*. Typhi to upregulate SPI-1 genes, resulting in invasion of the gallbladder epithelium. Flagellin allows *S*. Typhi to bind to gallstones and forms a scaffold to which further bacteria can bind. The extracellular matrix consists of curli fimbriae, Vi antigen, O-antigen and extracellular DNA.

Following the resolution of acute enteric fever, 2–5% of those infected with *S*. Typhi are thought to progress to an asymptomatic carrier state, where *S*. Typhi persists in the gallbladder and is intermittently shed in the stool (John *et al*. 2014) (Fig. 5). Both *S*. Typhi and *S*. Paratyphi A have been recovered at a high bacterial load from the gallbladders of patients undergoing cholecystectomy in Nepal (Dongol *et al*. 2012). The presence of bile induces transcriptional changes in *S*. Typhi, resulting in upregulation of the anti-oxidative enzymes superoxide dismutase and catalase by a mechanism dependent on quorum sensing (Walawalkar, Vaidya and Nayak 2016). Bile also induces *S*. Typhi, but not *S*. Typhimurium, to upregulate SPI-1 genes, increasing invasion of the gallbladder epithelium (Byrne *et al*. 2018). Interestingly, while typhoidal *Salmonella* serovars invade the gut without inducing neutrophil infiltration, *Salmonella-*positive (24 *S*. Typhi, 22 *S*. Paratyphi A and 2 *S. enterica* group C) gallbladders from cholecystectomy patients had a greater rate of neutrophil infiltration than culture-negative or non-*Salmonella* culture-positive gallbladders (Dongol *et al*. 2012).

Gallstones are a major risk factor for chronic carriage, affecting an estimated 90% of carriers (Lovane *et al*. 2016). Despite carriers tending to be asymptomatic, chronic carriage of *S*. Typhi and *S*. Paratyphi A each induce distinct plasma metabolome signatures, although the significance of this is unclear and has not yet been validated in an independent cohort (Näsström *et al*. 2018). Both *S*. Typhi and *S*. Typhimurium form biofilms on the surface of cholesterol gallstones in the presence of bile, giving rise to a thick, loosely packed cell matrix connected by a web of proteins, polysaccharides and extracellular DNA (Adcox *et al*. 2016). This process is dependent on both quorum sensing, allowing the bacteria to sense their population density, and flagellae, which allow attachment to the gallstone and provide a scaffold to which other bacteria can bind (Prouty, Schwesinger and Gunn 2002; Crawford *et al*. 2010). Biofilms can be directly visualised by electron microscopy on the surface of gallstones from human *S*. Typhi carriers, and are thought to render the bacteria resistant to antibiotic treatment (Crawford *et al*. 2010).

Although the Vi antigen is not necessary for biofilm formation, it does constitute part of the extracellular biofilm matrix (Adcox *et al*. 2016). Among those infected with *S*. Typhi, carriers constitute the few who raise substantial Vi antibody responses (Dougan and Baker 2014). The typhoid toxin, a multi-subunit exotoxin that induces cell cycle arrest (Galán 2016), does not play an obvious role in acute disease (Gibani *et al*. 2019), but may instead play a role in chronic disease. Transgenic expression of the typhoid toxin by *S*. Typhimurium results in development of a long-term asymptomatic infection in the liver following murine challenge (Del Bel Belluz *et al*. 2016).

As chronic carriers may act as a reservoir for infection, and therefore provide a barrier in the elimination of enteric fever, innovative strategies will be necessary to identify and treat carriers. At present, carriers are identified on the basis of Vi seropositivity, which has a low positive predictive value and will not be discriminatory in a vaccinated population, or bacterial shedding, which is intermittent (Näsström *et al*. 2018). The presence of a unique plasma metabolome signature in carriers presents a potential alternative avenue of diagnostics (Näsström *et al*. 2018). As *S*. Typhi biofilms are generally antibiotic resistant, carriers are currently treated by surgical removal of the gallbladder. Treatment of carriers with biofilm modulators, currently under development to treat hospital-acquired infections, might represent a less invasive alternative (Vila, Moreno-Morales and Ballesté-Delpierre 2020). As *S*. Typhi biofilm formation appears to be dependent on quorum sensing, the use of acyl-homoserine lactonases to disrupt these signals may also hold potential. Finally, if the typhoid toxin emerges as a major player in chronic disease, monoclonal antitoxins could be an attractive treatment.

## CONCLUSION

Typhoidal *Salmonella* serovars are characterised by human restriction, and an ability to evade immune detection and disseminate systemically. Binding specificity of the typhoid toxin and fimbriae to human cells may explain how *S*. Typhi is able to cause disease in humans, while iron restriction or detection by Rab32 may explain why *S*. Typhi is less adept at infecting non-human hosts. These insights into host restriction are contributing to the development of relevant animal models, which will accelerate preclinical development of vaccines and novel antimicrobials. Following invasion of the intestinal epithelium, while *S*. Typhi is able to evade detection by TLR4, the classical complement pathway and oxidative killing through production of the Vi capsule, adaptations in the LPS structure of *S*. Paratyphi A have enabled it to do the same. Knowledge of the virulence factors necessary to establish systemic disease presents an array of potential vaccine and therapeutic targets. While Vi-based vaccines have proved efficacious against *S*. Typhi, no vaccine is currently licensed against *S*. Paratyphi A. It is not currently known whether serovar replacement following widespread *S*. Typhi vaccination is a valid concern, but regardless it is likely that *S*. Paratyphi A will be responsible for a greater proportion of enteric fever cases in future. As such, development of a bivalent vaccine would be hugely beneficial to public health in Asia, where the two infections are co-endemic. This review also highlights several virulence factors shared with *S*. Typhimurium, potential targets of bi- or trivalent vaccines against invasive *Salmonella* disease in Africa. Furthermore, as extensively drug-resistant infections rise, novel therapeutic strategies will be needed to treat infections. The pathogenesis of *S*. Paratyphi B- and C-mediated enteric fever remains a mystery, but currently presents a less pressing global health concern. Despite a chiefly unicellular lifestyle, typhoidal *Salmonella* is able to form a multicellular community on the surface of gallstones and persist long term in the host. However, it is still unclear whether chronic infection has more far-reaching effects on host immunity than inducing gallbladder inflammation, or which bacterial virulence factors are key for colonisation. Innovative methods in diagnosing and treating chronic carriers will be key in the elimination of enteric fever.

While it has long been known that *S*. Typhi infection induces incomplete immunity, the past decade has revealed a myriad of ways by which *S*. Typhi evades the human immune response. With the availability of new vaccine programmes to control the disease, there will be a substantial impact on human health, but understanding of the biology of immune evasion will be essential to ensure eventual elimination of enteric fever from the world.
